# Perceived environmental barriers and facilitators of refugee children’s physical activity in/around refugee accommodation: a qualitative case study in Berlin

**DOI:** 10.1186/s13690-022-00993-1

**Published:** 2022-11-23

**Authors:** Siqi Chen, Martin Knöll

**Affiliations:** Urban Design and Planning Unit (UDP), Department of Architecture, Technische Universität Darmstadt, Darmstadt, Germany

**Keywords:** Migrants, Refugee facilities, Active play, Urban design, Barriers, Built environment, Meso environment, micro environment, Macro environment, Safety

## Abstract

**Background:**

Previous research have identified built environmental attributes associated with refugee children’s physical activity (PA); however, there is a lack of research focusing on refugee children’s environmental perceptions at the individual level. We examined the perceived environmental barriers and facilitators of refugee children’s PA.

**Methods:**

Perceptions of PA environments by refugee children (*n* = 15, 6 to 13 years old) and their parents (*n* = 10) were captured by questionnaires and drawing workshops from one refugee accommodation in Berlin. Besides, photovoice was conducted with three children to obtain an in-depth understanding of their experiences of existing environments for PA. Research was applied between June and July 2019. All research material was transcribed and analysed using thematic analysis.

**Results:**

Refugee children and their parents identified micro-environments as the centre of children’s daily PA, they usually played indoors but most parents perceived there was no spaces. In meso environments, children and parents thought there were insufficient spaces and were worried about neighbourhood safety. Furthermore, parents concerned more about ‘space accessibility’ for their children’s playing purposes instead of ‘space quality (e.g., equipment)’ . Children also indicated the importance of informal spaces for their PA.

**Conclusions:**

Refugee children perceive a lack of space and safety when attempting to play in the existing micro and meso environments. Related practitioners should focus on providing more play spaces in micro environments and safe access to existing neighbourhood playfields. These efforts can augment much-needed research on strategies to better integrate refuge facilities to their urban context and essential in minimising current health and spatial inequality issues these vulnerable groups face across Germany and worldwide.

**Supplementary Information:**

The online version contains supplementary material available at 10.1186/s13690-022-00993-1.

## Background

Germany is one of the countries hosting the biggest number of refugee children globally; 6.5% of the asylum seekers arrive in Berlin, and more than one-third are minors [1]. Those refugee children often spend a considerable amount of time in refugee accommodations [2, 3]. Studies have shown that refugee children have cramped living arrangements [4, 5], lack ‘dedicated spaces’ for play inside the camp [3], live in isolated and inaccessible city areas [6], or are worried about neighbourhood safety [4, 5]. Such environmental settings make it difficult for refugee children to engage in physical activity (PA), which is a fundamental determinant of health for children. It helps build a robust body, stable mental health and healthy relationships with peers [7–10]. The UNICEF report has shown that a large proportion of refugee children are not physically active [2]. Thus, it is possible that the built environment around refugee children hinders them from being active.

Reviews focus on built-environmental attributes associated with non-refugee children’s PA, such as availability/access of exercise equipment in micro-environments [11–14] and access to PA facilities (playgrounds, greenspaces), availability of sidewalks, neighbourhood perceived safety in meso-environments [15–19]. As mentioned, since refugee children live in very different environmental settings compared to non-refugee children, the existing findings of environmental attributes relevant to non-refugee children’s PA may not apply to refugee children. The authors’ previous review has identified that indoor and outdoor spaces in micro environments, formal/informal PA spaces, and neighbourhood safety in meso environments, are relevant to refugee children’s PA [20]. It also identified the research agenda and indicated gaps between existing built environments and refugee children’s PA. Previous research has presented findings on spatial characteristics and refugee children’s PA in multi-type Berlin-located refugee accommodations using quantitative and qualitative approaches [21, 22]. To gain a deeper understanding of refugee children’s PA; it is necessary to qualitatively identify environmental factors in relevant contexts associated with their PA at individual levels.

By assessing multi-ethnic, newcomer refugee children in one initial reception in Berlin, the authors sought to understand the perceived environmental barriers/facilitators of refugee children’s PA in/around their refugee accommodation.

### *Asylsystem* and initial reception in Berlin

**Fig. 1:**
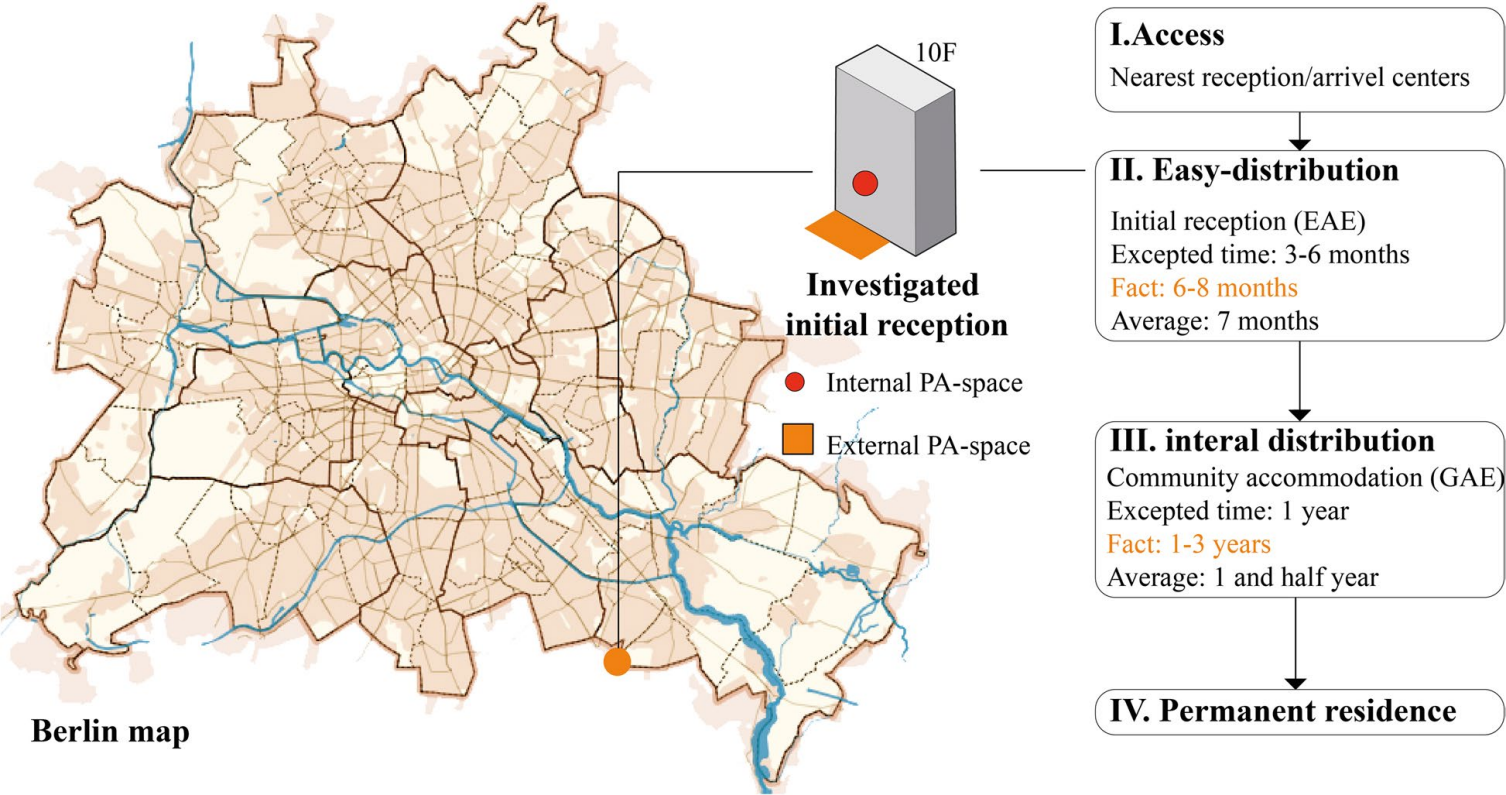
Current *Asylsystem* and investigated refugee accommodation in Berlin. Source: UNICEF, BAMF and State Office for Refugee Affairs Berlin (LAF) report

After asylum application submission, refugee families are designated to live in arriving centres or nearest available accommodations. They will then be distributed into initial receptions (Erstaufnahmeeinrichtung, EAE) as their first stations in Germany. After application evaluation, most families will be settled in community accommodation (Gemeinschaftsunterkünften, GAE). Private residences are possibly after leaving EAE (e.g., in Berlin) or specific evaluations, and differ between states [23]. Figure 1 illustrates *Asylsystem* and the investigated initial reception in Berlin.

### Micro, meso and macro environments

**Fig. 2:**
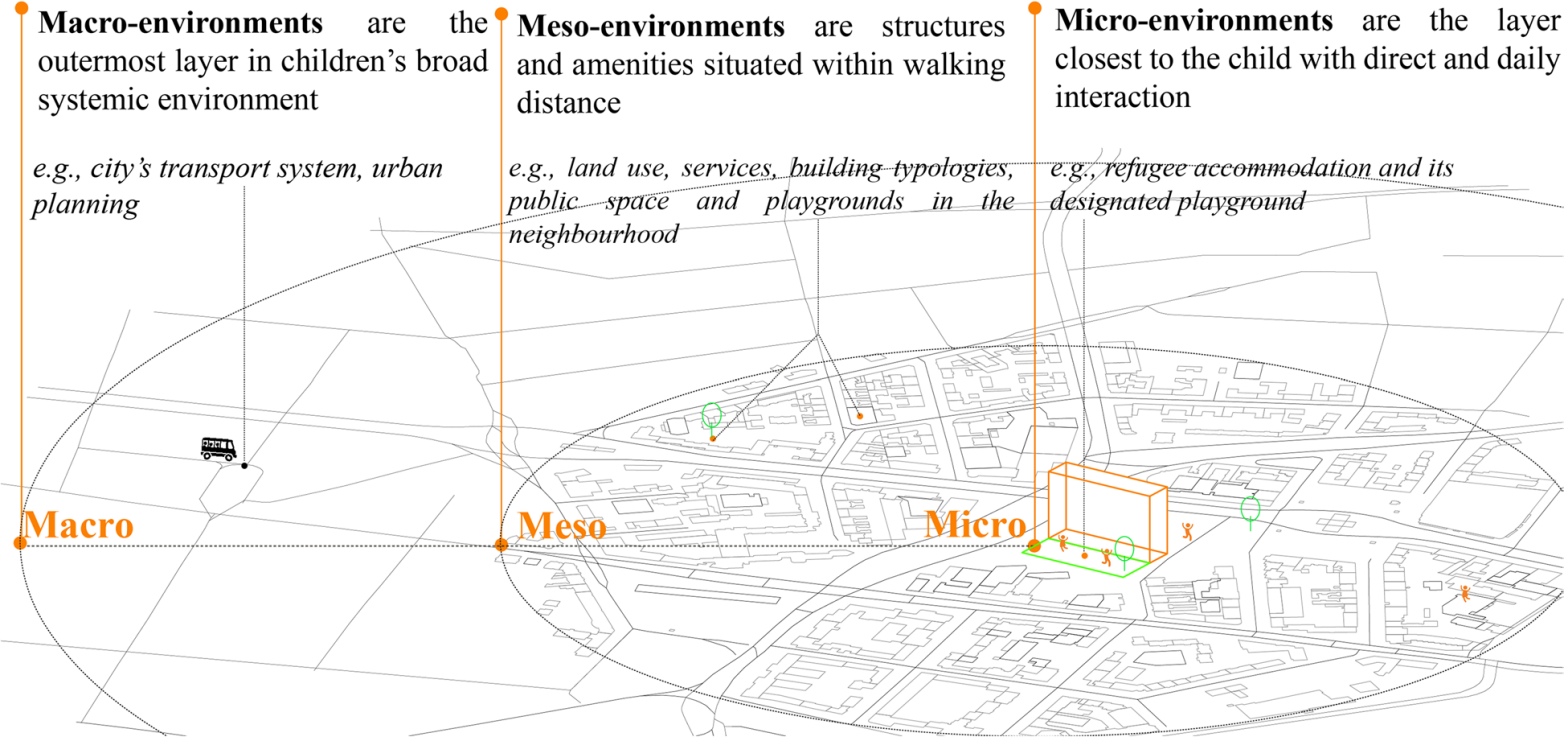
Diagram of environmental attributes on micro, meso and macro levels interacting with refugee children’s PA

Research rarely investigated refugee accommodations and their surroundings as individual built environment levels [24, 25]. Researchers have explored these *built environments* critically and tried to define the various nuances in the process. Bronfenbrenner’s ecological systems theory [26, 27] has been applied as a framework to understand refugee children’s day-to-day activities [28, 29] in this research. The built environment around refugee children includes three environmental layers of interest: *micro environment; meso environment,* and *macro environment*. The *micro environment* is the immediate vicinity of the child’s accommodation and contains the structures they directly contact in their daily lives [29]. Examples include the home/refugee camp and its designated playground [30]. The *meso environment* is the intermediate layer beyond the immediate surroundings but within the broader neighbourhood, including local schools, communities, streets and open spaces. The *macroenvironment* involves large-scale features of urban environments such as access to transport infrastructure and regional centres [31]. Dynamic and interactive interplay (such as PA behaviour) occur among all environment levels. The interaction of structure(s) within/between layer(s) is key to this theory. This research focuses on micro and meso environmental levels, as shown in Fig. 2.

### Formal and informal PA space in meso and macro environments

The authors’ previous review has distinguished and identified two types of spaces that are important for refugee children’s daily PA in built environments as ‘formal’ and ‘informal’ [20]. In this research, formal space is a play space/area constructed explicitly for the purpose of PA, including playgrounds and other sports fields [4, 5, 32, 33]. Previous qualitative studies reported that barriers to refugee children’s PA exist as limited or lack of access [4, 5] or lack of transportation to exercise facilities [33, 34].

Informal space is also essential for refugee children’s PA, including any urban spaces readily and freely available to refugee children. Examples as public open areas (see below Figure 7gh). Such spaces enable children to engage in being physically active, such as spontaneous play [3, 4, 35, 36].

### Perceived environments as keys to physical activity participation for refugee children

Abovementioned, built environments are essential for refugee children’s PA lives. Several researchers argued that environmental perceptions are often ignored in debates over refugee studies, but specific urban spaces are critical for refugees’ navigating experiences of displacement and resettlement [37–39]. Zeiher also noticed that some facilities were not truly/easily accessible for refugee children since they are often designed by adults [37]. Refugee children may be more cautious and sensitive about safety issues than non-refugee children [36] since they may have escaped from war situations or experienced military occupation [3], and they need to adapt to unfamiliar environments when they come to their host country. Such concerns by their parents are particularly salient, as where children can play is typically dictated by their parents [4], which were also mentioned by refugee accommodation staff in interviews [22]. Therefore, how refugee children (and their parents) perceive surrounding environments for playing (e.g., danger) is the key to their participation in PA. Methods.

### Context and setting

In light of the need for more studies on this topic specific to a particular setting, this explorative study using qualitative multi-methods sets out to understand what perceived environmental barriers and facilitators exist to PA among school-aged refugees (6–13 years old) residing in an initial reception in Berlin.

A further research aim was to investigate the feelings children perceived while moving around their everyday spaces and their perceptions of existing environments. As such, we paid close attention to the particulars of their experiences according to their identity [3]. The Unicef Report of Child Rights emphasises the freedom of children’s expressions, which encouraged us to apply more children-oriented methods in research [40]. The key to approaching children in research is to use techniques suitable for them, such as participant observation, task-based interviews, and creative methods [41]. Photography has revealed refugee children’s perspectives as arbiters of their own experience and allows them to document and perceive places that adult researchers often ignore [28, 42–44]. Literature also indicates that photography appears particularly prevalent when exploring different environmental levels among minority children [45]. Moreover, *Photovoice* [46] has been concluded as an appropriate communicative tool among children in marginalised situations [47].

The study design was conducted according to APA ethical guidelines concerning child protection reviewed [48] and approved by the Technical University of Darmstadt Ethics Committee (EK 26/2019) in June 2019.

### Participants

Non-probability sampling was used to recruit participants for this qualitative pilot study. Inclusion criteria were (1) not being diagnosed with physical or psychological diseases and (2) aged between 6 and 12 years old/attending primary schools. They were recruited via children department staff and researcher SC (a children volunteer) of the investigated accommodation. SC posted posters with their languages. Families were asked to provide consents while the children were carefully informed about the research aims and made aware that they were free not to answer/withdraw from the research at any time. In stage I, ten parents (Table 1) and fifteen children (Table 2) participated in June 2019. Three refugee children (RC2, 14 and 15) took part in stage II in June and July 2019, who were more engaged in stage I and willing to talk/share with the researcher SC.

**Table 1:** Demographic characteristics of refugee parent (RP) participants in stage I

Reference number	Countries of origin	Questionnaire language	Gender	Children’s number
RP1	Moldova	Russian	F	1
RP2	Iran	Persian/German	F	2
RP3	Moldova	Russian	M	2
RP4	Iran	Arabic	F	2
RP5	Iraq	Arabic	M	1
RP6	Iran	Persian	F	1
RP7	Moldova	Russian	F	1
RP8	Azerbaijan	Azerbaijani	F	2
RP9	Moldova	Russian	F	1
RP10	Iraq	Arabic	M	2

**Table 2:** Demographic characteristics of refugee children (RC) participants in stage I

Number	Countries of origin	Age	Gender	Parent (Table 1)
RC1	Moldova	6	M	RP1
RC2 (stage II)	Iran	10	F	RP2
RC3	Iran	8	F	RP2
RC4	Moldova	6	F	RP3
RC5	Moldova	6	F	RP3
RC6	Iran	6	F	RP4
RC7	Iran	9	M	RP4
RC8	Iraq	6	M	RP5
RC9	Iran	11	M	RP6
RC10	Moldova	6	F	RP7
RC11	Azerbaijan	11	M	RP8
RC12	Azerbaijan	13	F	RP8
RC13	Moldova	7	M	RP9
RC14 (stage II)	Iraq	7	F	RP10
RC15 (stage II)	Iraq	9	M	RP10

### Instruments and procedures

Our participatory approach encompassed a place-based method focused on children’s playing, which was conceptualised to include places and how they may mirror and shape relations of PA between children [3, 49]. Parents’ questionnaires and children’s workshops in stage I were formulated to understand their perception and children’s PA in micro and meso environments. Besides, a photovoice workshop in stage II was applied to gain in-depth insight into individuals’ experiences and deepen the qualitative approach. This research was designed with the help of experts and refugee accommodation staff. It aims to provide instruments (clock, drawing, camera) to refugee children, with which protect them from negative feelings about spaces, express their emotions in a non-judgmental/safe space, and discuss their ideas, concerns, and perspectives in an active and participatory way [40, 50, 51].

#### Stage I

Parents were asked to evaluate PA space accessibility/availability in their children’s existing micro and meso environments with a five-point scale questionnaire (see Additional file 1). The questionnaire ended with a filling content of their children’s detailed PA timeline. While parents answered, the children would finish the following workshops in 30 minutes.

The workshop started with a short questionnaire (see Additional file 2). Afterwards, children entered their PA information (where, when, activity type) by keywords or body language into a playable clock (see Additional file 3a). Children were also asked to ‘draw their play (the facilities/place/equipment they were playing in/with)’ in an A3 paper with defined environmental scales (micro: indoor and outdoor, meso: around the accommodation) as shown in Additional file 3b.

#### Stage II

Stage II was 3 days’ photographing of refugee children’s playing (where, what, their mood). Three children took part in with provided cameras Participants finished this independently without authors’ influence. SC represented printed photos on an A1 poster by correct timelines and environmental scales (see Additional file 4). Children were asked to put on different mood tags (see below Fig. 6b) and explain their feelings when taking photos in unstructured interviews.

### Data collection and analysis

The structured questionnaires in stage I were translated into six languages by professionals. German/English were used in oral communications and interpretations in stage II. All narrative materials from stage I/II were transcribed and translated by SC and a bilingual professional translator. Two previously mentioned tools worked as supplementary carriers that helped children express their feelings: (1) drawing and the clock, which reminded them of their PA perceptions on different scales; (2) mood tags, which helped to express their emotions related to spaces.

More demonstrative explanations and groups of quotations were reassembled into different themes using NVivo software [26, 37, 52]. SC reviewed materials from each participant, intending to analyse and determine naturally apparent codes inductively. MK and SC discussed apparent themes, agreed on the coding categories, and incorporated them into the final coding framework. Additional inputs were produced to interpret data for finalising the findings. Data were determined to reach theoretical saturation when no new themes/viewpoints/ keywords emerged from the materials [53]. This design allows possibilities for reading and backtracking.

### Researcher characteristics and reflexivity statement

This research is based in the Urban Design and Planning Unit (UDP), Department of Architecture, Technical University of Darmstadt, Germany. Built-environments related to refugee children’s health behaviours (e.g., PA) in Germany are not only understudied, but much of its first empiric material concerning individual refugee child’s level is challenging to approach. The UDP’s mission is to deliver empirical data and evidence-based strategies to inform the much-needed transformation towards more healthy and inclusive cities. SC had lived in Berlin for 4 years at the time of the study, and she had been a children’s volunteer in the investigated accommodation since April 2017. She is a non-Arabic speaker but an experienced social worker for communication. MK is an expert in Urban Design and Health and the research supervisor.

## Result

### Results of stage I

#### Insufficient formal PA spaces

**Fig. 3:**
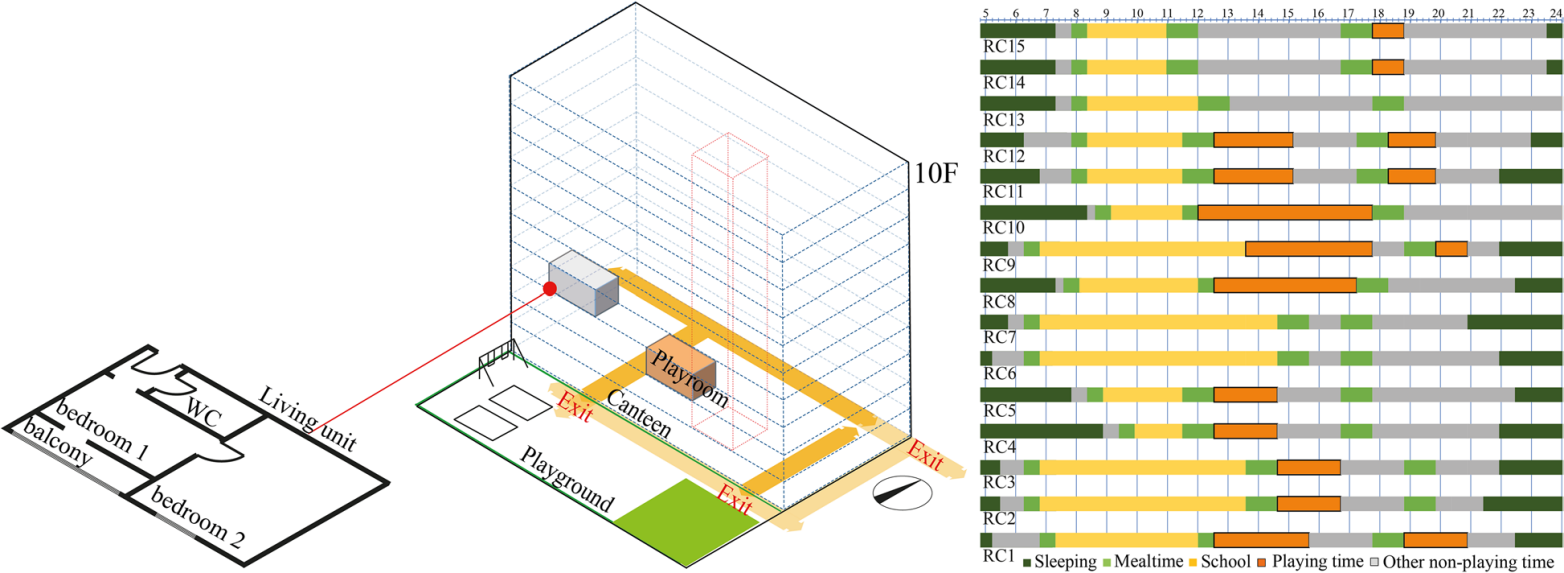
(**a**) living unit example; (**b**) diagram of PA spaces in the micro environment; (**c**) daily PA timelines of 15 refugee children

This accommodation was a former hotel; all interviewed families settled in two rooms living units with a balcony (Fig. 3a). A playground was set outside the canteen with equipment (e.g., swing, Fig. 3b) at level 0. There was one indoor playroom on level 2, opening by schedule on workdays. This accommodation was located at the boundary of Berlin with poor public transportation (see Fig. 1). Fig. 3c illustrates children’s daily playing lives: after breakfast and school (8:00 to 13:00/14:00), children gathered in the playroom/playground until dinnertime (1 to 2 hours). Time spent after dinner was individual (maybe a shorter period for playing), and then they went to bed. Children/parents reported children’s PA mostly happened inside the accommodation (playroom or playground).

#### The importance of informal PA spaces

**Fig. 4:**
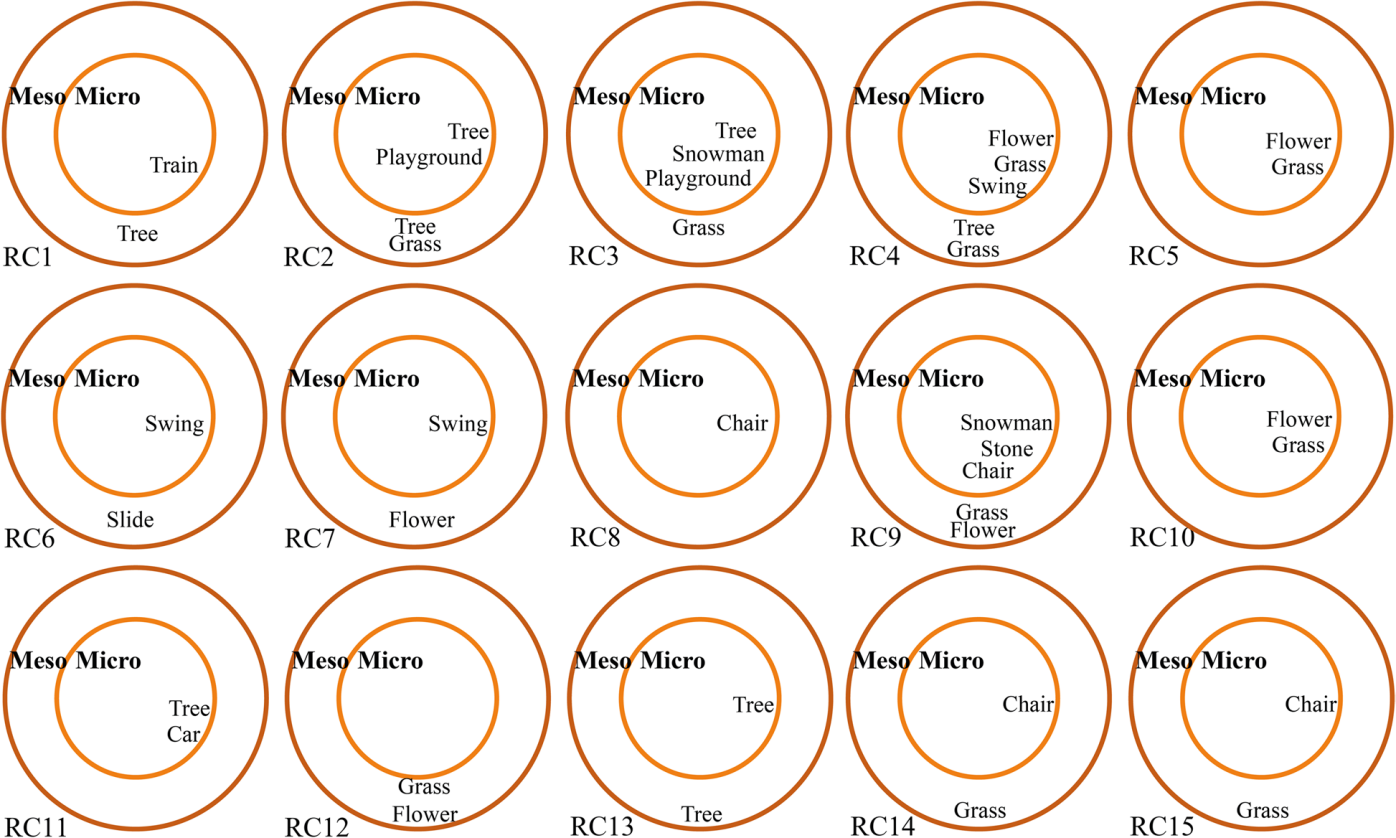
Categories of children’s drawing (related to PA and space) in micro and meso environments

**Fig. 5:**
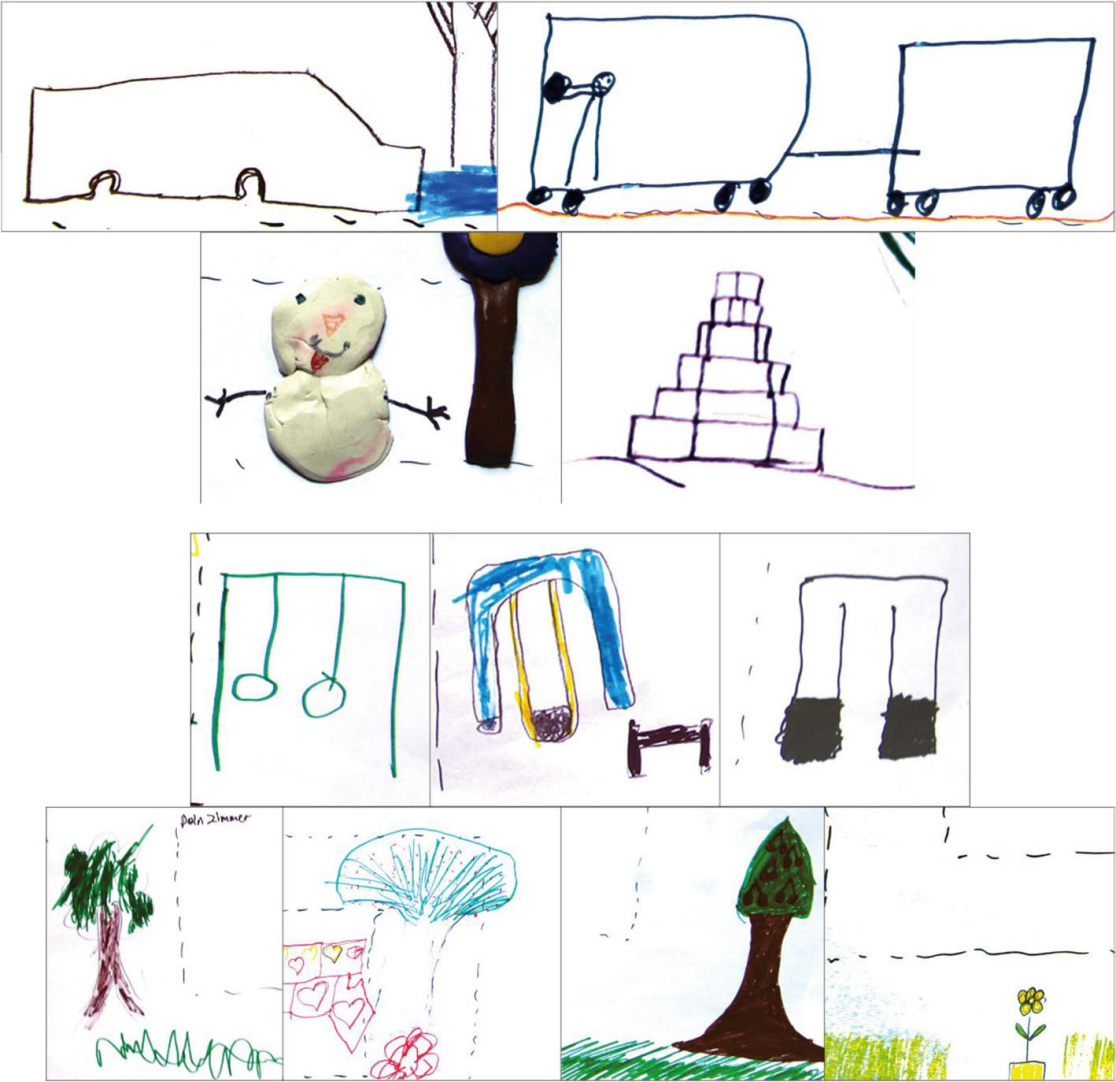
drawings of (**a**) playgrounds with PA equipment; (**b**) transportation to PA spaces; (**c**) snowman and Stone Jenga playing; (**d**) informal PA spaces as nature

As shown in Fig. 4, in the micro environment, only three children drew a swing for their daily playing (Fig. 5a), one had no impressions of playing and the other two reported they needed transportation for outdoor playing (Fig. 5b).

In meso environments, 11/15 children had impressions of playing, and one identified slide as their play equipment. The drawings also depicted informal activity not governed by formal regulations but creative, such as ‘making snowman’ or ‘Stone Jenga’ (Fig. 5c). 10/15 children described informal places for their activities, such as greenspace/unstructured spaces for playing (Fig. 5d).

#### Parents environmental perceptions

Table 3 summarises ten parents’ perspectives of environments for their children’s PA. In the micro environment, seven parents thought there was not enough space in the playroom. As for the playground, one parent marked it as no space for playing, and six parents thought it was too small. In summary, most parents thought there was no accessible play space in the micro environment.

**Table 3:** Ten parents’ perspectives of existing micro and meso environments for children’s PA

*Micro environment*	
**You find there is (see options as below) space in this building for your children’s playing (e.g., playroom):**	
No space	3/10
Too small	4/10
Enough space	3/10
**You find there is (see options as below) space in the playground beside the building for your children’s playing:**	
No space	1/10
Too small	6/10
Enough space	2/10
*Meso environment*	
**You find there is (see options as below) space in parks /small playgrounds around the building for your children’s playing:**	
No space	5/10
Too small	3/10
Enough space	1/10
**Where (e.g., on the way to school) do your children like to stay in the neighbourhood?**	
Park nearby	2/10
**Do you think the neighbourhood is safe?**	
Yes	1/10
No	2/10
Not sure	7/10 (do not go out)

In the meso environment, all but one parent thought there were no accessible/limited spaces for children’s playing. Two parents thought the neighbourhood was unsafe, while seven parents were unsure about neighbourhood safety because they did not go out very often.

### Results of stage II

#### Environmental perception of PA space

**Fig. 6:**
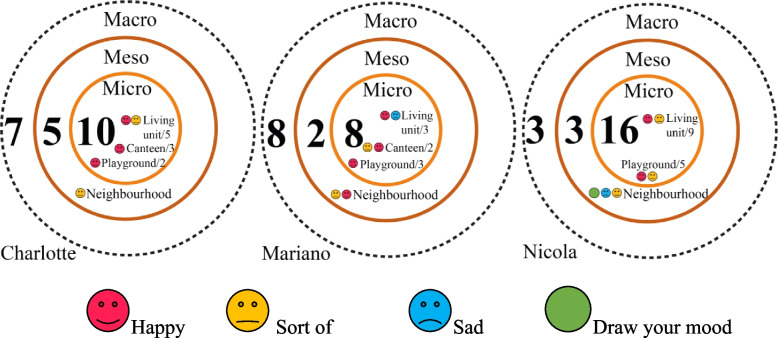
(**a**) Perceived photos of three children by three environmental levels; (**b**) mood tag examples

Figure 6a illustrates the discrete photography spaces of three environmental layers children perceive. The micro environment was where they took most photos and spent most daily PA. They usually stayed indoors in a non-satisfied mood and felt happy when they were playing in the playground. They used positive words for PA behaviour but negative words to define the micro environment. Children took the fewest photos with negative descriptions of the meso environment. They took highly abstracted photos and used mainly natural expressions of the macro environment.

#### Daily PA timeline and patterns

##### Charlotte [7] and Mariano [9]^1^,^2^

**Fig. 7:**
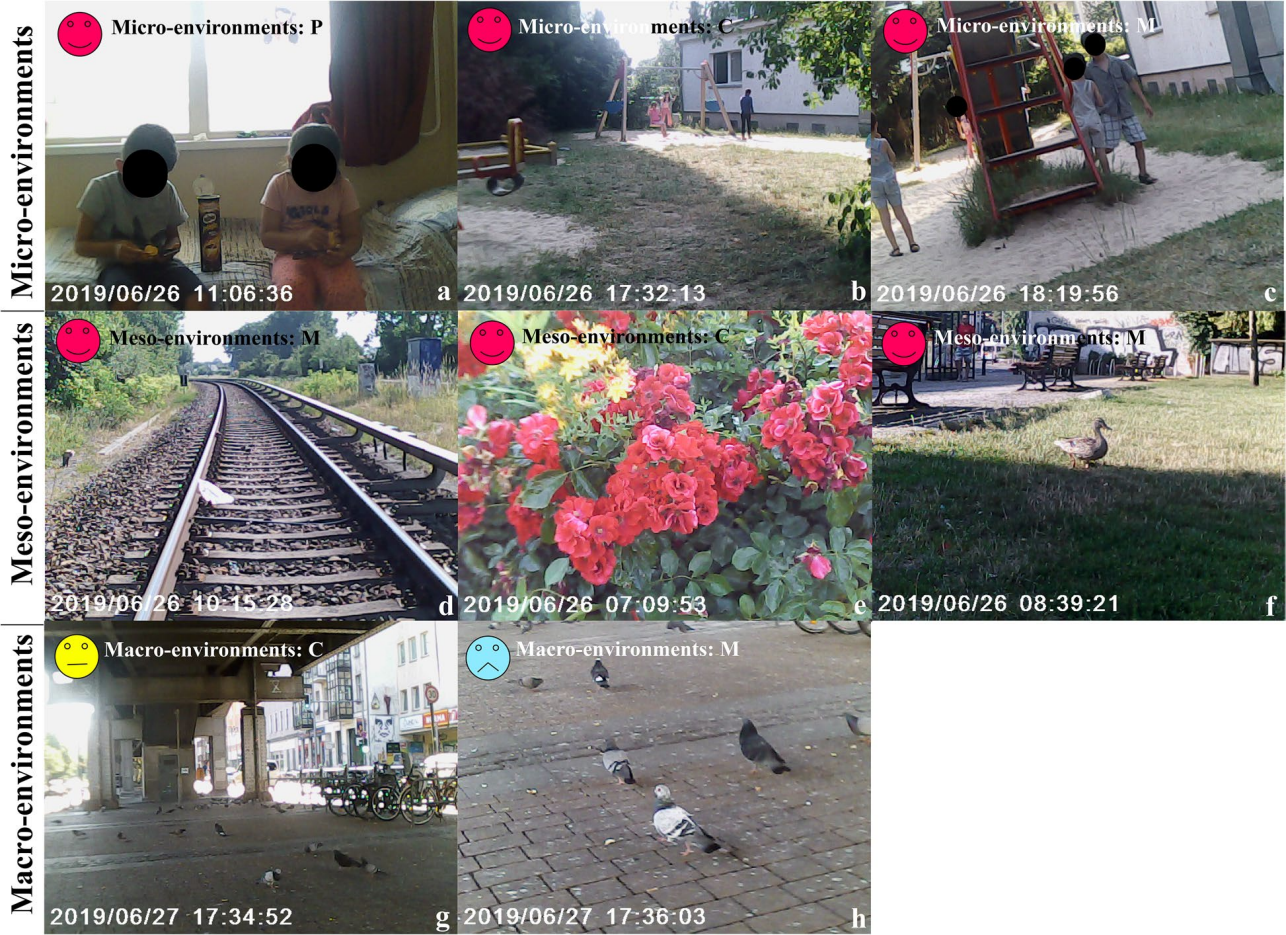
Charlotte and Mariano’s perceived photos of three environmental levels concerning their PA. P: photo by parents under children requests; C: photo by Charlotte; M: photo by Mariano

Charlotte and Mariano took transportation for morning play by crossing an abandoned railway since there was no immediate play area around this neighbourhood (Fig. 7d). The two siblings liked to chase each other on the grassland (Fig. 7ef). They watched time for leaving to catch the served lunch. In the afternoon, two children played characters inside their living unit (Fig. 7a). They mentioned their father preferred them to play indoor under supervision. Most children gathered in the playground after dinner (Fig. 7bc). The author asked if they had a fixed group or time for playing:

Charlotte (translated by her father): “No, Mariano or my father move the swing for me, sometimes I play with Nicola and her sister (they speak the same language), but I don’t know their room number.”

Mariano (translated by his father): “No, I play with Charlotte. I think the things in the playground are too childish.”

They regarded open areas under bridges as playing spaces (Fig. 7g). Mariano said his parents did not like this place because of the danger (Fig. 7h).

##### Nicola [10]^3^

**Fig. 8:**
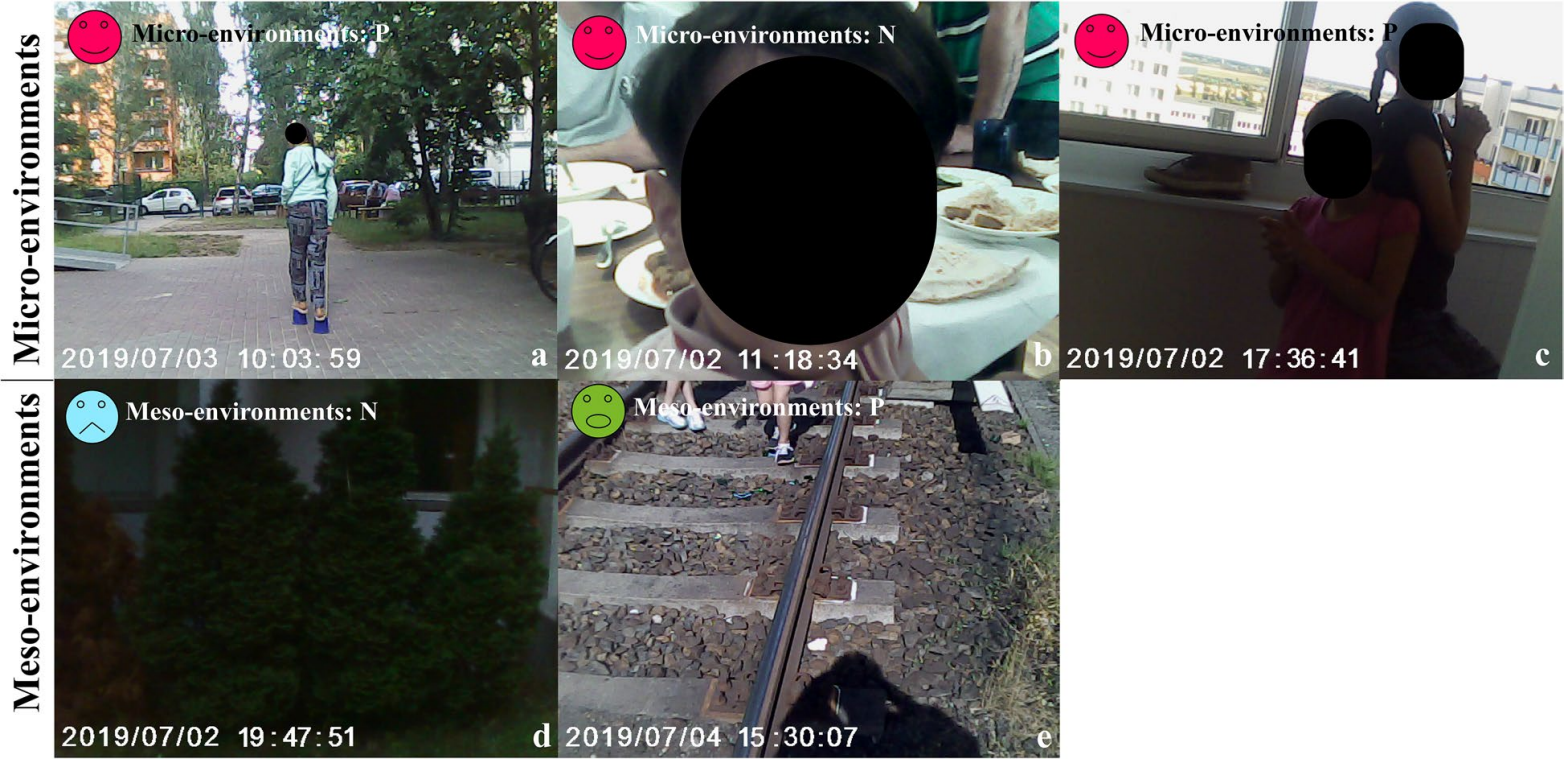
Nicola’s perceived photos of two environmental levels concerning her PA. P: photo by parents under request; N: photo by Nicola

Nicola showed us her creative, informal activity of playing with cans in Fig. 8a. She could only play indoors or immediate neighbourhood since her parents had no time for supervision; sometimes, she needed to take care of younger residents (Fig. 8b):

“My (little) sister is (was) a gymnast in Iran”, they showed the author “, I’m also good at sport, I have good balance, we don’t have many things to play … (The swing and sand playground) are for small children … (she pointed to the photo). I made these myself” she put a *happy* mood tag on the photo 8a.

Recently, her favourite indoor activity before dinner was role play with her sister (Fig. 8c):

“We played like the movie; yes, 007, we imagined we are spies, so interesting, we chase each other in the room!”

She did not go to the playground very often; instead, she liked to explore the neighbourhood but was unhappy with the existing one because of scared trees (Fig. 8d) and abandoned railways (Fig. 8e); she also mentioned transportation was essential when playing outside the accommodation:

Nicola: “the scary trees in Fig. 8d,” she imitated a monster “the neighbourhood is cold … I smiled at other children (neighbourhood); they don’t (smile back), no other playground around here.”

“Dangerous, no place to play” she drew a *panic* mood tag and put it on Fig. 8e, “I asked my father to take the photo, but funny (to play on the train rail), I want somewhere else around here (to play).”

## Discussion

### Synthesis and interpretation

**Fig. 9:**
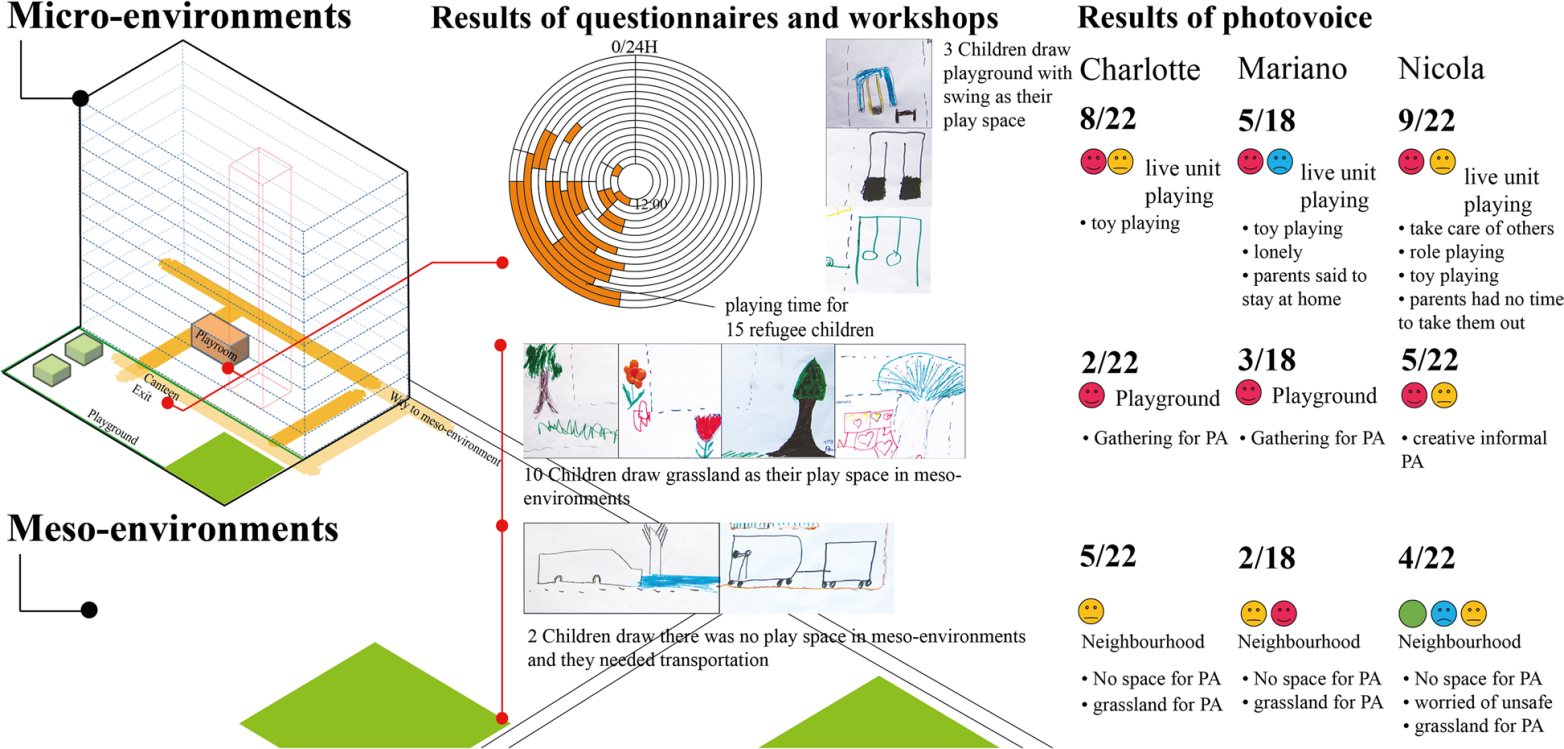
Summarisation of key findings of stages I and II

Figure 9 summarises the key findings: most parents thought there were neither enough indoor nor outdoor PA spaces in the micro environment, and the time children spent on PA was limited. Children indicated most of their PA happened indoors. They both identified refugee accommodation (micro environments) as the centre of children’s daily PA lives.

In meso environments, most parents thought there were not enough PA spaces (formal or informal) and worried about neighbourhood safety. In both stages, children identified informal spaces (as grassland) for their play and thought there were insufficient playing spaces.

Moreover, an interesting theme emerged from the material analysis: refugee parents paid less attention to the existing ‘quality aspects (e.g., size, PA equipment)’ of built environments since these might be formed differently from their countries of origin [4, 34, 36]. They cared more about if the PA spaces, either formal or informal, were available for their children’s playing purposes.

### Refugee children vs non-refugee children

**Fig. 10:**
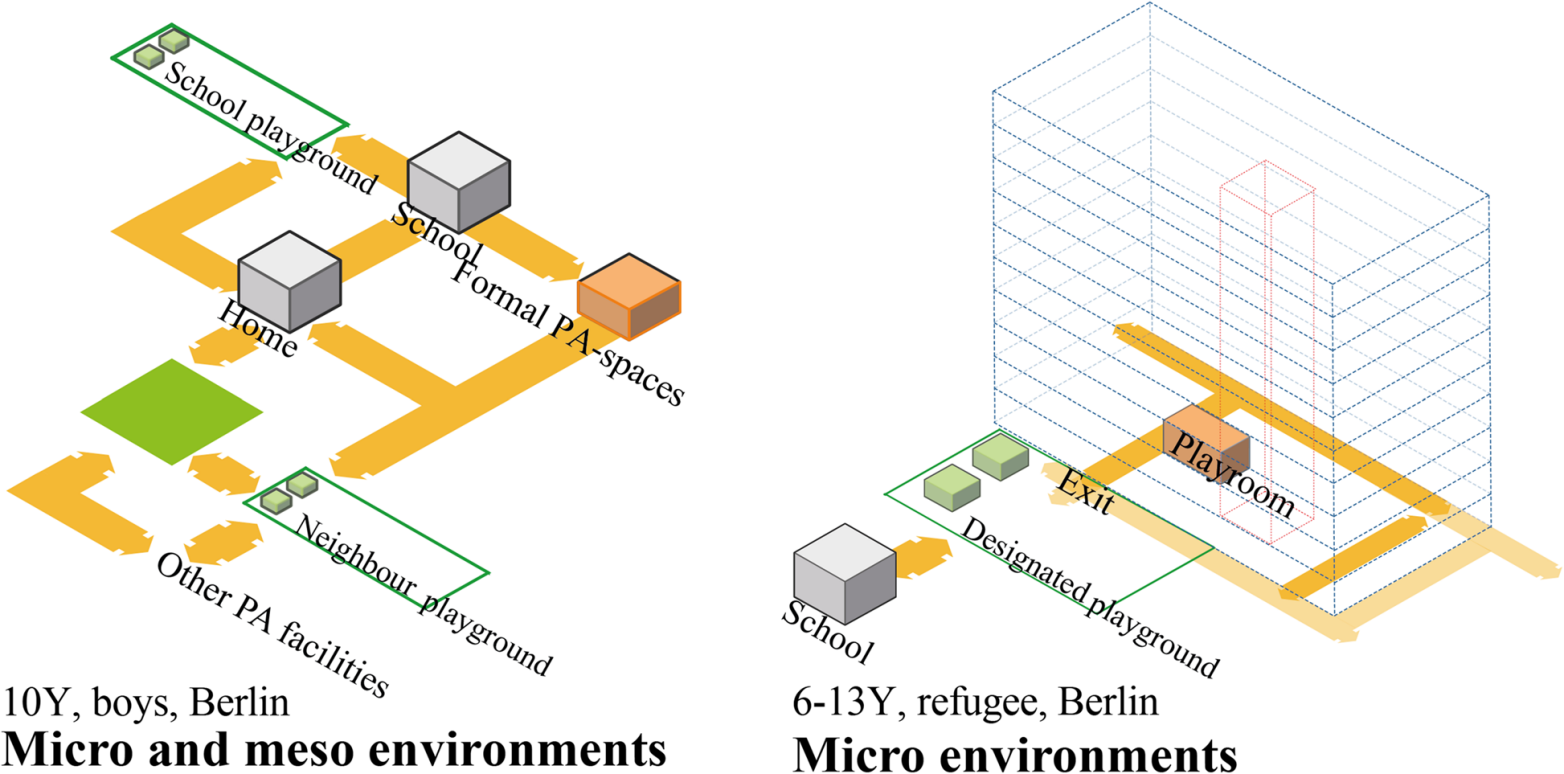
PA locations of (**a**)non-refugee and (**b**) refugee children in Berlin

A previous review established the research agenda of built environmental barriers and facilitators to physical activity for refugee children, i.e., access to physical activity facilities and neighbourhood safety, which were similar to those identified for non-refugee children’s PA [20]. However, the findings do not necessarily mean that refugee and non-refugee children have equal access to PA spaces. The comparable Fig. 10 of PA locations to non-refugee [37] and refugee children in Berlin shows us some interesting facts: refugee children’s PA mostly happens in micro environments, while non-refugee children’s PA happens in micro and meso environments. Future research needs to compare refugee and non-refugee children in terms of how active they are, where they engage in PA and the relations of this spatial equality. Such research would highlight the PA levels and disparities between refugee and non-refugee children. Considering that the location of refugee accommodation is a matter of the discretion of local authorities, future research on this topic is needed to inform where best to build refugee facilities to enhance refugee children’s activity, health and safety.

### Neighbourhood perceived safety

With regard to safety concerns, they are often about road safety or local crime for non-refugee children [16]. As mentioned above, they may be more cautious and sensitive about safety issues than non-refugees because of their previous experiences [36]. This research adds some new insight into how refugee children and parents perceive danger in surrounding environments: safety concerns by their parents are particularly salient, as children’s play locations are supervised/decided by their parents. Children’s photos provided additional evidence that most photos were taken indoors, where their parents preferred their children to play, and all outdoor images were under supervision. Future research needs to investigate how refugee children and parents perceive danger in meso environments deeply; moreover, if it differs from non-refugee children and parents.

### Formal vs informal spaces for refugee children’s PA

Refugee children may have limited access to ‘formal PA spaces’ for many reasons: they may find neighbourhoods with limited facilities due to locations [4, 54]; the neighbourhood may be regarded as unsafe to get to these spaces, existing facilities might already be ‘occupied’ by local children [55], or facilities with existing PA programs for children might be not affordable for refugee children [5, 56–58]. Some studies also exposed that it is difficult for children and their parents who live in short-term accommodations to make plans or take advantage of formal PA spaces because they are not in a stable living situation [54, 59, 60]. This research found that children and parents had negative impressions of ‘formal PA spaces’; besides, they had lower expectations of these spaces and outdoor equipment styles based on the experiences from their countries of origin.. Most children talked about the enjoyable PA environment they played before.

For all of those barriers, informal spaces for PA become very important for refugee children as a hidden agenda: in line with Hordyk [55], children from our research explained how they made the best of the limited access to nature they had, like describing the games they played, noting details such as birds tracking and flowers flourishing. Compare to formal, naturally informal spaces may be more familiar playfields to newcomer refugee children since the global similarity of nature [58]. Furthermore, the findings from this study can be explained by Hertting and Karlefors: refugee children enjoy sporting activities in informal places since activity could be agreed upon by rules from participants but not governed by formal regulations [35]. However, the importance of informal space suggested in our findings may reflect the lack of opportunities to participate in formal PA spaces. Given that it can be challenging to organise sports in refugee settings, it is vital that informal spaces exist where children can be active with peers during leisure time. Diverse opportunities are essential for refugee children’s PA, whether in formal or informal spaces. Future studies can assess the effect and feasibility of PA targeting refugee children in both spaces.

### Strengths and limitations of the research

There were several limitations due to the explorative nature. This qualitative research had a small sample size; therefore, a restriction for the volume of data collection within the research scope. It was a rather tricky task to approach refugee children and their families in Berlin: most accommodations refused participation without giving any reason; families were not willing to collaborate; trust work issues; language barriers, and cultural sensibility. Thus, the study site and samples were participants willing to collaborate in this research, which may raise the issue of whether this case was truly representative. However, the analysis from this study provides insights into the relationship between perceived environmental barriers/facilitators and refugee children’s PA.

As mentioned before, language barriers existed as the authors were non-Arabic native speakers. Limited command of a language may lead one to say what one’s command allows rather than what one wants to say [42]. Participants had problems understanding/expressing their feelings. On the other hand, the first author SC was also a volunteer (where the data collection took place), families might have been more likely to report certain aspects due to higher trust. These findings could be considered rarely empirical materials that contributes to the knowledge of refugee children’s PA in existing built environments.

In line with previous research [29, 42], photovoice in stage II was applied as a tool for deepening individual PA experience of children: they chose photos they wanted to discuss, and the authors learned about their perceptions. There may be an argument raised from this children-oriented research design: on the one hand, the photos helped them express their perceptions at different environmental levels, which adult researchers may ignore. On the other hand, children may become more physically active compared to their daily standards. The camera might work as a PA catalyst instead of a recording object, which motivated them to take more vivid photos. The fact, all of the children performed the task with significant commitment; they represented the photographs as ‘experts’ on their living conditions.

## Conclusion

This qualitative study provides a better understanding of perceived environmental barriers and facilitators of refugee children’s PA in/around refugee accommodations. Refugee children perceived environmental barriers (e.g., not enough indoor/ outdoor spaces) and potential facilitators (e.g., informal PA spaces) when attempting to engage in physical activity in existing micro and meso environments. Refugee parents did not know of existing play spaces in micro nor meso environments, or, in the majority of the cases did not perceive them as sufficiently in size and in safety. The findings may serve as a starting point for related practitioners to understand refugee children’s health conditions at individual levels, to optimise the gap of existing built-environments spatial limitations (e.g., provide safe access to neighbourhood playfields) and potentialities (e.g., identifying informal space). These efforts are essential in minimising current health and spatial inequality issues these vulnerable groups face across Germany and worldwide.

## Supplementary Information

**Additional file 1.** Questionnaire for parents of their children’s daily playing (English version)*.

**Additional file 2.** Questionnaire for children in stage I (English version).

**Additional file 3.** (a) playable clock poll example for children in stage I; (b) environmental scale paper example for children’s drawing in A3 paper.

**Additional file 4.** A1 poster example for unstructured interviews in stage II (process material).

## Data Availability

All available data generated or analysed during this study are included in this published article and its Additional files.

## Abbreviations

PA: Physical activity
EAE: Erstaufnahmeeinrichtungen (initial reception)
GAE: Gemeinschaftsunterkunften (community accommodation)
UNICEF: United Nations High Commissioner for Refugees
BAMF: Bundesamt für Migration und Flüchtlinge (Federal Office for Migration and Refugees)
LAF: Landesamt für Flüchtlingsangelegenheiten Berlin (State Office for Refugee Affairs Berlin)
RP: Refugee parents
RC: Refugee children

## Footnotes

1 Photo taking and video recording conducted 26th to 28th June 2019; Interview conducted 1st July 2019.

2 The names here were fictitious names

3 Photo taking and video recording conducted 2nd to 4th July 2019; Interview conducted 8th July 2019.
