# Supplementary material for: Perceived environmental barriers and facilitators of refugee children’s physical activity in/around refugee accommodation: a qualitative case study in Berlin
Source: Arch Public Health. 2022 Nov 23;80:242. doi: 10.1186/s13690-022-00993-1 (PMC9686116; doi:10.1186/s13690-022-00993-1)
Supplement: Supplementary file 1 — Additional file 1. Questionnaire for parents of their children’s daily playing (English version)*. [file 13690_2022_993_MOESM1_ESM.docx]

Additional file1: Questionnaire for parents of their children’s daily playing (English version)*

*Persian, Azerbaijani, Russian, German and Arabic versions are available from the corresponding author on reasonable request

*The demographic situation*

1. **How many children do you have?**
2. **Who do you think your children are willing to play with (possible for more than one option)?**

☐ peers (other children in the facility)

☐ Sisters or brothers

☐ Children care department or other volunteers

*The existing environment of the accommodation*

1. **You find there is (see options as below) space in this building for your children’s playing (e.g., playroom):**

☐ no space ☐ too small ☐ either too big or too small ☐ enough space ☐ too big

1. **You find there is (see options as below) space beside this building for your children’s playing (e.g., playground):**

☐ no space ☐ too small ☐ either too big or too small ☐ enough space ☐ too big

1. **You find there is (see options as below) space in parks/playgrounds for your children’s playing:**

☐ no space ☐ too small ☐ either too big or too small ☐ enough space ☐ too big

1. **How long do your children play outside every day?**

☐less than half an hour ☐less than one hour ☐ 1 to 2 hours ☐ more

*The neighbourhood*

1. **Where (e.g. on the way to school) do your children like to stay in the neighbourhood? (Please show it on the map)?**
2. **Do you think the neighbourhood is safe?**

☐yes ☐no ☐not sure

1. **Do you think the neighbourhood is friendly?**

☐yes ☐no ☐not sure

*Everyday life conditions*

*A typical day for your children*

1. **Could you help me to describe the timeline of your children every day (Please fill it with the time range number, for example, 10 to 10:30)**


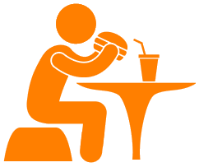

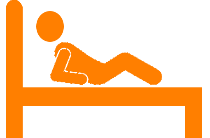


**☐ Wake up**


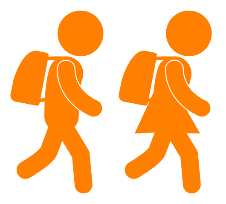
**☐ Breakfast**


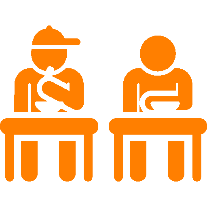


**☐ School**

**☐ Lunch**

**☐ Afternoon playing**


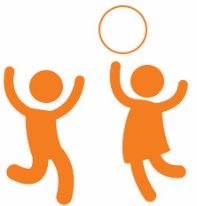

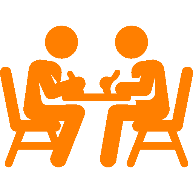
**☐ Afternoon tea**

**☐ Workshop**

**☐Dinner**


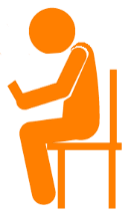


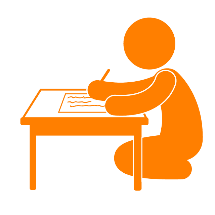
**☐ Evening playing**

**☐ Phone time**

**☐Homework**


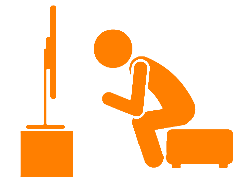
**☐ Housework**


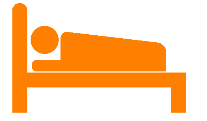
**☐ TV**

**☐ Go to bed**

**Thank you so much for helping!**
