# Supplementary material for: Perceived environmental barriers and facilitators of refugee children’s physical activity in/around refugee accommodation: a qualitative case study in Berlin
Source: Arch Public Health. 2022 Nov 23;80:242. doi: 10.1186/s13690-022-00993-1 (PMC9686116; doi:10.1186/s13690-022-00993-1)
Supplement: Supplementary file 2 — Additional file 2. Questionnaire for children in stage I (English version). [file 13690_2022_993_MOESM2_ESM.docx]

Additional file2: Questionnaire for children in stage I (English version)

1. ***Tell us yourself***


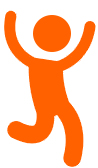

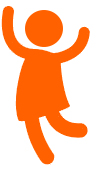


- 1. **Are you ☐**Girl **☐** Boy **☐** No idea
  2. **How old are you?**

**☐**6 **☐**7  **☐**8 **☐**9 **☐**10  **☐**11 **☐**12

- 1.
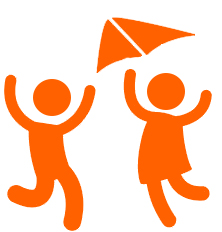
**What is your favourite activity/sport?**
  2. **Whom do you like to play with (possibly more than one option)?)?**

**☐** Peers (other children) **☐** my sisters or brothers

**☐** Parents **☐**voleenter and children staff

**☐** Others:

***Your day (With the o‘clock game)***

**2.1 What are you doing? And where (put it on the clock)?**
