# Supplementary material for: Perceived environmental barriers and facilitators of refugee children’s physical activity in/around refugee accommodation: a qualitative case study in Berlin
Source: Arch Public Health. 2022 Nov 23;80:242. doi: 10.1186/s13690-022-00993-1 (PMC9686116; doi:10.1186/s13690-022-00993-1)
Supplement: Supplementary file 3 — Additional file 3. (a) playable clock poll example for children in stage I; (b) environmental scale paper example for children’s drawing in A3 paper. [file 13690_2022_993_MOESM3_ESM.docx]

Additional file 3: (a) playable clock poll example for children in stage I; (b) environmental scale paper example for children’s drawing in A3 paper


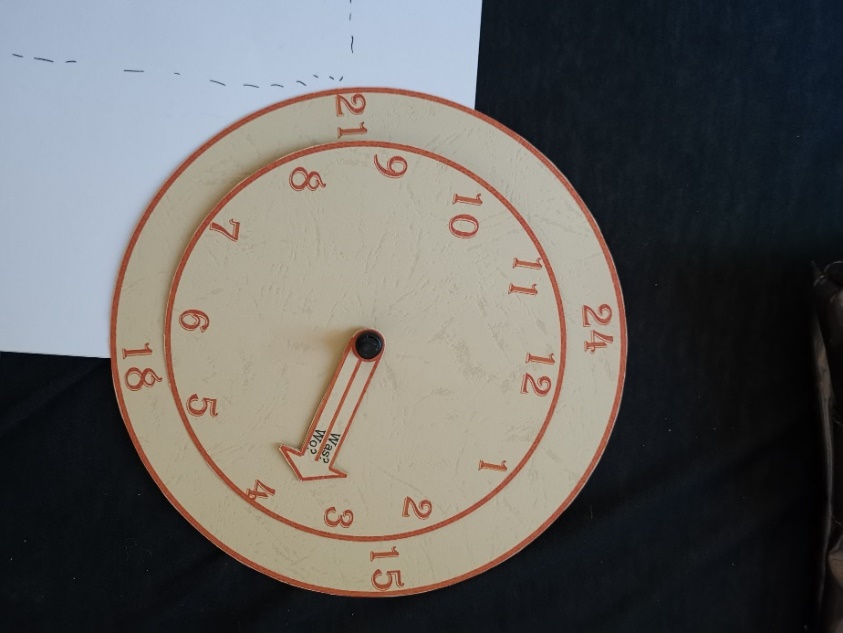
*
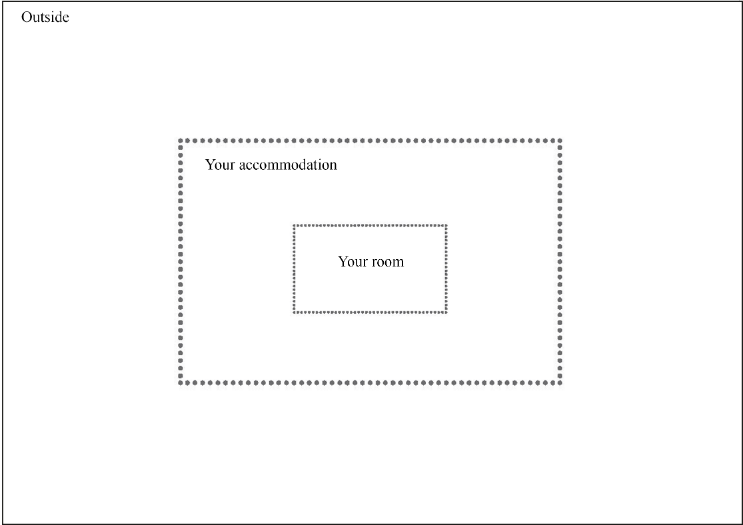
*
