# Supplementary figures and images for: Perceived environmental barriers and facilitators of refugee children’s physical activity in/around refugee accommodation: a qualitative case study in Berlin
Source: Arch Public Health. 2022 Nov 23;80:242. doi: 10.1186/s13690-022-00993-1 (PMC9686116; doi:10.1186/s13690-022-00993-1)

Additional file 4: A1 poster example for unstructured interviews in stage II (process material);


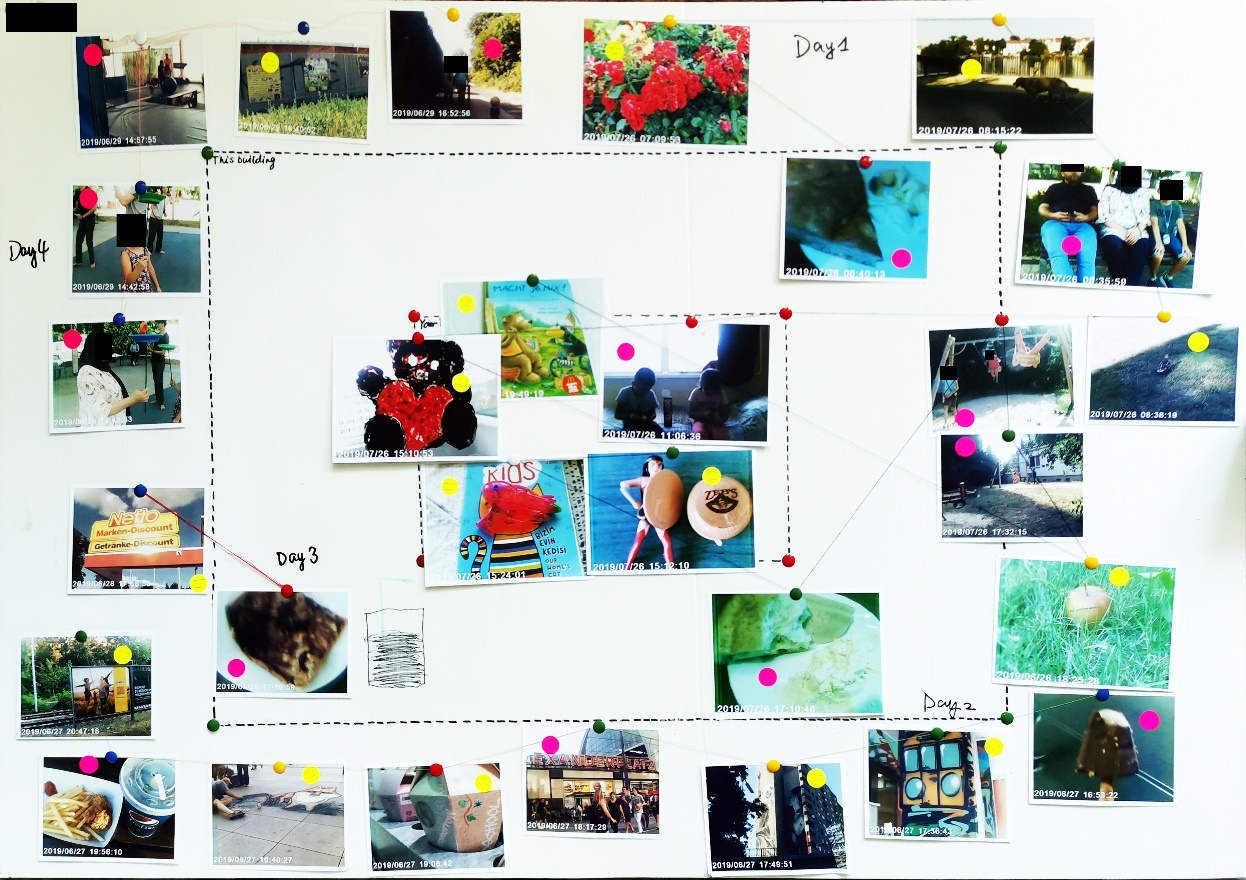

Supplement: Supplementary file 4 — Additional file 4. A1 poster example for unstructured interviews in stage II (process material). [file 13690_2022_993_MOESM4_ESM.docx]
